# Mechanistic and Structural Insights Into the Unique TetR-Dependent Regulation of a Drug Efflux Pump in *Mycobacterium abscessus*

**DOI:** 10.3389/fmicb.2018.00649

**Published:** 2018-04-05

**Authors:** Matthias Richard, Ana Victoria Gutiérrez, Albertus J. Viljoen, Eric Ghigo, Mickael Blaise, Laurent Kremer

**Affiliations:** ^1^CNRS UMR 9004, Institut de Recherche en Infectiologie de Montpellier, Université de Montpellier, Montpellier, France; ^2^Unité de Recherche, Microbes, Evolution, Phylogeny and Infection, Institut Hospitalier Universitaire Méditerranée Infection, Marseille, France; ^3^Centre National de la Recherche Scientifique, Campus Joseph Aiguier, Marseille, France; ^4^Institut National de la Santé et de la Recherche Médicale, Institut de Recherche en Infectiologie de Montpellier, Montpellier, France

**Keywords:** *Mycobacterium abscessus*, TetR regulator, MmpL, efflux pump, structure, thiacetazone analogs, EMSA

## Abstract

*Mycobacterium abscessus* is an emerging human pathogen causing severe pulmonary infections and is refractory to standard antibiotherapy, yet few drug resistance mechanisms have been reported in this organism. Recently, mutations in *MAB_4384* leading to up-regulation of the MmpS5/MmpL5 efflux pump were linked to increased resistance to thiacetazone derivatives. Herein, the DNA-binding activity of MAB_4384 was investigated by electrophoretic mobility shift assays using the palindromic sequence IR_S5/L5_ located upstream of *mmpS5/mmpL5*. Introduction of point mutations within IR_S5/L5_ identified the sequence requirements for optimal binding of the regulator. Moreover, formation of the protein/IR_S5/L5_ complex was severely impaired for MAB_4384 harboring D14N or F57L substitutions. IR_S5/L5_*/lacZ* reporter fusions in *M. abscessus* demonstrated increased β-galactosidase activity either in strains lacking a functional MAB_4384 or in cultures treated with the TAC analogs. In addition, X-ray crystallography confirmed a typical TetR homodimeric structure of MAB_4384 and unraveled a putative ligand binding site in which the analogs could be docked. Overall, these results support drug recognition of the MAB_4384 TetR regulator, alleviating its binding to IR_S5/L5_ and steering up-regulation of MmpS5/MmpL5. This study provides new mechanistic and structural details of TetR-dependent regulatory mechanisms of efflux pumps and drug resistance in mycobacteria.

## Introduction

*Mycobacterium abscessus* is a rapid growing mycobacterium (RGM) that has recently become an important health problem (Mougari et al., 2016). This non-tuberculous mycobacterial (NTM) pathogen can cause serious cutaneous, disseminated or pulmonary infections, particularly in cystic fibrosis (CF) patients. In CF patients, infection with *M. abscessus* is correlated with a decline in lung function and poses important challenges during last-resort lung transplantation (Esther et al., 2010; Smibert et al., 2016). An epidemiological study has recently documented the prevalence and transmission of *M. abscessus* between hospital settings throughout the world, presumably *via* fomites and aerosols and uncovered the emergence of dominant circulating clones that have spread globally (Bryant et al., 2016). In addition, *M. abscessus* exhibits innate resistance to many different classes of antimicrobial agents, rendering infections with this microorganism extremely difficult to treat (Nessar et al., 2012; van Dorn, 2017). Recent studies have started to unveil the basis of the multi-drug resistance characterizing *M. abscessus*, uncovering a wide diversity of mechanisms or regulatory networks. These involve, for example, the induction of the *erm(41)* encoded 23S rRNA methyltransferase and mutations in the 23S *rRNA* that lead to clarithromycin resistance (Nash et al., 2009), the presence of a broad spectrum β-lactamase that limits the use of imipenem (Dubée et al., 2015; Lefebvre et al., 2016, 2017) or the presence of *eis2*, encoding an acetyl-transferase that modifies aminoglycosides, specifically induced by *whiB7*, which contributes to the intrinsic resistance to amikacin (Hurst-Hess et al., 2017; Rominski et al., 2017b). Other studies reported the role of the ADP-ribosyltransferase MAB_0591 as a major contributor to rifamycin resistance (Rominski et al., 2017a) whereas MAB_2385 was identified as an important determinant in innate resistance to streptomycin (Dal Molin et al., 2018).

Recently, we reported the activity of a library of thiacetazone (TAC) derivatives against *M. abscessus* and identified several compounds exhibiting potent activity against a vast panel of clinical strains isolated from CF and non-CF patients (Halloum et al., 2017). High resistance levels to these compounds were linked to mutations in a putative transcriptional repressor MAB_4384, together with a strong up-regulation of the divergently oriented adjacent locus encoding a putative MmpS5/MmpL5 transporter system. That ectopic overexpression of MmpS5/MmpL5 in *M. abscessus* also increased the minimal inhibitory concentration (MIC) to analogs of TAC further suggested that these two proteins may act as an active efflux pump which was sufficient to confer drug resistance (Halloum et al., 2017). In addition to uncovering new leads for future drug developments, this study also highlighted a novel mechanism of drug resistance in *M. abscessus*. Unexpectedly, an important difference relies on the fact that, in *M. tuberculosis*, MmpS5/MmpL5 acts as a multi-substrate efflux pump causing low resistance levels to antitubercular compounds such as clofazimine, bedaquiline, and azoles (Milano et al., 2009; Andries et al., 2014; Hartkoorn et al., 2014) whereas the *M. abscessus* strains overexpressing MmpS5/MmpL5 are very resistant to the TAC analogs but fail to show cross-resistance against clofazimine or bedaquiline (Halloum et al., 2017). This implies that, despite their high primary sequence identity, the MmpS5/MmpL5 orthologs from *M. tuberculosis* and *M. abscessus* do not share the same substrate specificity. Moreover, whereas Rv0678, the cognate regulator of MmpS5/MmpL5 in *M. tuberculosis* belongs to the MarR family (Radhakrishnan et al., 2014), MAB_4384 is part of the TetR family of regulators and the change in the transcriptional level of *mmpS5/mmpL5* was much more pronounced in the *M. abscessus* mutants than in the *M. tuberculosis* mutants (Milano et al., 2009; Hartkoorn et al., 2014).

The TetR transcriptional regulatory factors are common single component signal transduction systems found in bacteria. These proteins possess a conserved helix-turn-helix (HTH) signature at the N-terminal of the DNA-binding domain as well as a ligand binding domain (LBD) located at the C-terminal part (Cuthbertson and Nodwell, 2013). They often act as repressors and interact with a specific DNA target to prevent or abolish transcription in the absence of an effector. In contrast, the binding of a specific ligand to the LBD induces structural changes, conducting the dissociation of the repressor from the target DNA, and the subsequent transcription of the TetR-regulated genes. Being largely associated with resistance to antibiotics and regulation of genes coding for small molecule exporters, TetR regulators also govern expression of antibiotic biosynthesis genes, quorum sensing and in distinct aspects in bacterial physiology/virulence (Cuthbertson and Nodwell, 2013). A recent global analysis indicated that the TetR regulators represent the most abundant class of regulators in mycobacteria, the vast majority remaining uncharacterized (Balhana et al., 2015). In order to provide new insight into the mechanism of gene regulation by TetR regulators in mycobacteria and to describe a new and specific drug resistance mechanism in *M. abscessus*, we focused our efforts on the molecular and structural characterization of the MAB_4384-dependent regulation of MmpS5/MmpL5.

In this study, a combination of genetic and biochemical analyses was applied to determine the specificity of this regulatory system in *M. abscessus* and to describe the contribution of key residues that are important in driving the DNA-binding of MAB_4384 to its operator. We report also the crystal structure of the MAB_4384 TetR regulator. Overall, the results provide new insights into the regulation of members of the MmpL family and on a novel mechanism of drug resistance in *M. abscessus*.

## Materials and Methods

### Plasmids, Strains, Growth Conditions, and Reagents

The *Mycobacterium abscessus* subsp. *abscessus* CIP104536^T^ reference strain and all derived mutant strains are listed in Supplementary Table S1. Strains were grown in Middlebrook 7H9 broth (BD Difco) supplemented with 0.05% Tween 80 (Sigma-Aldrich) and 10% oleic acid, albumin, dextrose, catalase (OADC enrichment; BD Difco) (7H9^T/OADC^) at 30°C or in Sauton’s medium in the presence of antibiotics, when required. On plates, colonies were selected either on Middlebrook 7H10 agar (BD Difco) supplemented with 10% OADC enrichment (7H10^OADC^) or on LB agar. For drug susceptibility testing, cultures were grown in Cation-Adjusted Mueller-Hinton Broth (CaMHB; Sigma-Aldrich). The TAC analogs D6, D15, and D17 were synthesized as reported previously (Coxon et al., 2013) and dissolved in DMSO. Other antibiotics were purchased from Sigma-Aldrich.

### Cloning of Wild-Type and Mutated MAB_4384 and Site-Directed Mutagenesis

*MAB_4384* was PCR-amplified from *M. abscessus* CIP104536^T^ purified genomic DNA using the *MAB_4384_full* primers (Supplementary Table S2) and Phusion polymerase (Thermo Fisher Scientific). The amplicon was cloned into pET32a restricted with KpnI and HindIII (New England Biolabs), enabling the introduction of *MAB_4384* in frame with the thioredoxin and poly-histidine tags as well as a Tobacco Etch Virus protease (TEV) cleavage site between the N-terminus of MAB_4384 and the tags. The *MAB_4384* alleles harboring the g40a (D14N) and t169c (F57L) mutations were PCR-amplified using the primers described above and using the purified genomic DNA of the two spontaneous resistant *M. abscessus* strains to TAC analogs reported previously (Halloum et al., 2017). The double mutant carrying both g40a and t169c mutations was obtained from the *MAB_4384* (g40a) allele using the PCR-driven primer overlap extension method (Aiyar et al., 1996). Briefly, two separate PCR reactions were set up using Phusion polymerase. The first was generated using the forward primer *MAB_4384_full* Fw and a reverse internal primer *MAB_4384_DM* Rev harboring the nucleotide substitution responsible for the mutation. The second PCR was set up with an internal forward primer *MAB_4384_DM* Fw overlapping the internal reverse primer *MAB_4384_DM* Rev and with the original reverse primer *MAB_4384_full* Rev. PCR products were purified, annealed and amplified by a last PCR amplification with the *MAB_4384_full* primers. All mutated genes were cloned into pET32a, as described for wild-type *MAB_4384*.

### Expression and Purification of MAB_4384 Variants

The various pET32a-derived constructs containing either the wild-type or the mutated *MAB_4384* gene alleles were used to transform *Escherichia coli* strain BL21 Rosetta 2 (DE3) (Novagen). Cultures were grown in Luria-Bertani (LB) medium containing 200 μg/mL ampicillin and 30 μg/mL chloramphenicol until an optical density at 600 nm (OD_600_) of between 0.6 and 1.0 was reached. Liquid cultures were then placed on icy water for 30 min prior to the addition of 1 mM isopropyl β-D-1-thiogalactopyranoside (IPTG) and incubation for an additional 20 h at 16°C. Bacteria were then collected by centrifugation (6,000 × *g*, 4°C, 60 min) and the pellets were resuspended in lysis buffer (50 mM Tris-HCl pH 8, 200 mM NaCl, 20 mM imidazole, 5 mM β-mercaptoethanol, 1 mM benzamidine). Cells were opened by sonication and the lysate clarified by centrifugation (28,000 × *g*, 4°C, 45 min) and subjected to a first step of nickel affinity chromatography (IMAC) (Ni-NTA Sepharose, GE Healthcare Life Sciences). After elution, the protein was dialyzed overnight at 4°C in a buffer containing 50 mM Tris-HCl pH 8, 200 mM NaCl, 5 mM β-mercaptoethanol and TEV protease (1 mg of protease/50 mg of total protein) to cleave the thioredoxin and histidine tags from the recombinant proteins. The dialyzed preparations were purified again by IMAC, followed by an anion exchange chromatography step (HiTrap Q Fast Flow, GE Healthcare Life Sciences) as well as a final polishing step using size exclusion chromatography (SEC) (Sephadex^TM^ 75 10/300 GL, GE Healthcare Life Sciences) and a buffer containing 50 mM Tris-HCl pH 8, 200 mM NaCl and 5 mM β-mercaptoethanol.

The selenomethionine-substituted protein was expressed in the methionine auxotroph *E. coli* strain B834 (DE3) (Novagen). A 1L culture was grown very densely in LB medium containing 200 μg/mL ampicillin for 36 h at 37°C. Bacteria were harvested by centrifugation and the pellets resuspended in minimal medium A without antibiotic and methionine traces (M9 medium, trace elements, 0.4% glucose, 1 μM MgSO_4_, 0.3 mM CaCl_2_, biotin and thiamine at 1 μg/mL). After an additional wash in medium A, the bacterial pellet was resuspended in 6L of medium A containing 200 μg/mL ampicillin and incubated for 2 h at 37°C. Finally, S/L selenomethionine was added at a final concentration of 100 μg/mL. After 30 min of incubation, expression of the protein was induced with 1 mM IPTG for 5 h at 37°C. The protein was purified using a protocol similar to the one used for the proteins expressed in the *E. coli* strain BL21 Rosetta 2(DE3).

### Determination of Oligomeric States of MAB_4384 by Size Exclusion Chromatography

The oligomeric state of MAB_4384 and MAB_4384:DNA complex in solution were assessed on an ENrich^TM^ SEC 650 size exclusion column (Bio-Rad) run on an ÄKTA pure 25M chromatography system (GE Healthcare Life Science). The protein, DNA or protein:DNA complex were eluted with 50 mM Tris-HCl pH 8, 200 mM NaCl and 5 mM β-mercaptoethanol at a flow rate of 0.4 mL/min at 4°C. MAB_4384 (dimer) was concentrated to 3.9 mg/mL, while DNA was at 2.8 mg/mL and complexes were formed at different protein(dimer)/DNA molar ratios of 1:1, 2:1, 3:1. The molecular weights were determined based on a calibration curve generated using the Gel Filtration Markers Kit (Sigma-Aldrich) for proteins ranging from 12,400 to 200,000 Da. The column void volume was assessed with the elution peak of dextran blue. The apparent mass was obtained by plotting the partition coefficient *K*_av_ against the log values of the molecular weights of the standard proteins.

### Disruption of *MAB_4384* and *mmpL5* in *M. abscessus*

To generate *MAB_4384* and *mmpL5* knock-out mutants, internal fragments of the genes were PCR-amplified using Phusion polymerase and the specific oligonucleotide sets: *MAB_4384*::pUX1 Fw with *MAB_4384*::pUX1 Rev and *mmpL5*::pUX1 Fw with *mmpL5*::pUX1 Rev, respectively, digested with NheI and BamHI and ligated to NheI-BamHI-linearized pUX1 (Supplementary Table S1), a suicide vector specifically designed to perform gene inactivation in *M. abscessus* (Viljoen et al., 2018). Electrocompetent *M. abscessus* was transformed with the plasmids pUX1-*MAB_4384* and pUX1-*mmpL5* and plated on 250 μg/mL kanamycin LB plates. After 5 days of incubation at 37°C, red fluorescent colonies were selected and gene disruption resulting from homologous recombination between the plasmid DNA and the target genes was confirmed by PCR and sequencing with appropriate primers (Supplementary Table S2).

### Quantitative Real-Time PCR

Isolation of RNA, reverse transcription and qRT-PCR were done as reported earlier (Halloum et al., 2017) using the primers listed in Supplementary Table S2.

### Drug Susceptibility Assessment

The MICs were determined according to the CLSI guidelines (Woods et al., 2011), as reported earlier (Halloum et al., 2017).

### Electrophoretic Mobility Shift Assays

First, a typical DNA binding motif recognized by the TetR regulator, often composed of palindromic sequences or inverted repeats, was identified *in silico* within the intergenic region located between *MAB_4384* and the *MAB_4383c (mmpS5_Mabs_)/MAB_4382c (mmpL5_Mabs_)* gene cluster, hereafter referred to as IR_S5/L5_, using the MEME Suite 4.20.0 online tool^1^ (Bailey et al., 2009). A 45 bp double stranded DNA fragment (Probe 1) containing the 27 bp palindromic sequence was labeled with fluorescein at their 5′ ends (Sigma-Aldrich). Increasing amounts of purified MAB_4384 protein were co-incubated with 280 nM of the fluorescein-labeled probes in 1X Tris Base/acetic acid/EDTA (TAE) buffer for 1 h at room temperature. The samples were then subjected to 6% native polyacrylamide gel electrophoresis for 30 min at 100 V in 1X TAE buffer. Gel shifts were visualized by fluorescence using an Amersham Imager 600 (GE Healthcare Life Sciences). All additional modified probes listed Supplementary Table S2 and used in this study were synthetized and used in electrophoretic mobility shift assay (EMSA) assays, as described above.

### Construction of β-Galactosidase Reporter Strains and β-Gal Assays

The *lacZ* reporter gene encoding the β-galactosidase was amplified from the *E. coli* HB101 using primers listed in Supplementary Table S2. The amplicon was cloned into pMV261 cut with BamHI and HindIII, thus yielding pMV261_P_*hsp*60__*lacZ*. The 208 bp intergenic region IR_S5/L5_ was amplified by PCR using *M. abscessus* CIP104536^T^ genomic DNA and the *MAB_4384_*P_S5/L5_ primers (Supplementary Table S2) and subsequently cut with XbaI and BamHI. The *hsp60* promoter was removed from pMV261_P_*hsp*60__*lacZ* construct by restriction using XbaI and BamHI and replaced with IR_S5/L5_, thus creating pMV261_P_S5/L5__*lacZ*. A promoterless pMV261_*lacZ* construct was also generated by removing the *hsp60* promoter from the pMV261_P_*hsp*60__*lacZ* with BamHI and XbaI, blunting the overhang extremities using the T4 DNA Polymerase and self-religation.

The β-galactosidase activity of the *M. abscessus* strains carrying either wild-type or mutated *MAB_4384* alleles and the various β-gal reporter constructs was monitored streaking the strains directly on 7H10^OADC^ agar plates supplemented with 100 μg/mL kanamycin and 50 μg/mL X-gal (Sigma-Aldrich). The quantification of the β-gal activity was also assayed in liquid medium using a protocol adapted from Miller’s method. Briefly, a 10 mL culture in Sauton’s medium supplemented with 0.025% tyloxapol was grown until the OD_600_ reached 0.6–1. Cultures were collected by centrifugation (4,000 × *g* for 10 min at 4°C) and the bacterial pellets were resuspended in 700 μL 1X phosphate-buffered saline (PBS) prior to mechanical lysis by bead beating (3 min treatment, 30 Hz). Lysates were finally centrifuged at 16,000 × *g* for 10 min at 4°C. 10 μL of clarified lysate were co-incubated 30 min at 37°C with 100 μL of reaction buffer (60 mM Na_2_HPO_4_, 40 mM NaH_2_PO_4_, 10 mM KCl, 1 mM MgSO_4_, 50 mM β-mercaptoethanol) in 96-well plates. Enzymatic reactions were initiated by adding 35 μL of 2-Nitrophenyl β-D-galactopyranoside (ONPG, Sigma-Aldrich) at 4 mg/mL and absorbance was recorded at 420 nm at 34°C using a Multimode Microplate Reader POLARstar Omega (BMG Labtech). The β-galactosidase specific activity (SA_β-*Gal*_) was calculated using the following formula: SA_β-Gal_ = (Absorbance_420nm_ × min^-1^)/(OD_280nm_ × liter of culture). To test the β-gal-induction by the TAC analogs, the drugs were added directly to the cultures grown in Sauton’s medium (OD_600_ = 0.6–1) and incubated with slow shaking for 96 h at 37°C. The β-gal activity was determined as described above.

### Crystallization, Data Collection, and Refinement

The MAB_4384 crystals were grown in sitting drops in MR Crystallization Plates (Hampton Research) at 18°C by mixing 1.5 μl of protein solution concentrated to 4.7 mg/mL with 1.5 μl of reservoir solution made of 100 mM sodium cacodylate pH 6.5, 200 mM MgCl_2_, 16% PEG 8000 and 5% DMSO. Crystals were briefly soaked in 100 mM Cacodylate buffer pH 6.5, 200 mM MgCl_2_, 16% PEG 8000, 5% DMSO and 10% PEG 400 prior to being cryo-cooled in liquid nitrogen. The selenomethionine-substituted MAB_4384 crystals were obtained in sitting drops in 96-well SWISSCI MRC plates (Molecular Dimension) at 18°C by mixing 0.8 μl of protein solution concentrated to 2.5 mg/mL with 0.8 μl of reservoir solution consisting of 35% (v/v) 1,4 dioxane. Crystals were cryo-cooled without any additional cryo-protection. Data were processed with *XDS* and scaled and merged with *XSCALE* (Kabsch, 2010). Data collection statistics are presented in **Table 1**. The MAB_4384 structure was solved by the single wavelength anomalous dispersion method. *AutoSol* from the *Phenix* package was used to solve the structure (Adams et al., 2010). Twelve of the fourteen potential selenium sites in the asymmetric unit were found using a resolution cutoff of 3.4 Å for the search of the Se atoms. After density modification, a clear electron density map for the two TetR monomers allowed initial model building. The resulting partial model was used to perform molecular replacement with the 1.9 Å native dataset using *Phaser* (McCoy et al., 2007) from the *Phenix* package (Adams et al., 2010). *Coot* (Emsley et al., 2010) was used for manual rebuilding while structure refinement and validation were performed with the *Phenix* package (Adams et al., 2010). The statistics for data collection and structure refinement are displayed in **Table 1**. Figures were prepared with PyMOL^2^. The atomic coordinates and the structure factors for the reported MAB_4384 crystal structure has been deposited at the Protein Data bank (accession number 5OVY).

**Table 1:** Data collection and refinement statistics.

	MAB_4384 native	Selenium peak
**Data collection statistics**		
Beamline	ESRF-ID30B	ESRF-ID30B
Wavelength (Å)	0.979	0.979
Resolution range (Å)	36.5–1.9 (1.96–1.9)	47–2.3 (2.38–2.3)
Space group	P 1 21 1	P 1 21 1
Unit cell (Å,°)	40.8 100.8 56.0 90 105.8 90	41.4 99.3 55.7 90 106.9 90
Total reflections	73028 (7378)	126590 (12013)
Unique reflections	31705 (3193)	18983 (1849)
Multiplicity	2.3 (2.3)	6.7 (6.5)
Completeness (%)	92.1 (93.4)	98.6 (98.1)
Mean I/sigma (I)	11.09 (1.06)	11.66 (1.36)
Wilson B-factor (Å^2^)	33.9	46.02
R-meas	0.06 (0.92)	0.13 (1.21)
CC1/2	0.99 (0.51)	0.97 (0.67)
CC^∗^	1 (0.82)	0.99 (0.89)
**Data refinement statistics**		
Reflections used in refinement	31695 (3193)	
Reflections used for R-free	2000 (201)	
R-work	0.184 (0.312)	
R-free	0.213 (0.351)	
Number of non-H atoms	3460	
Macromolecules	3178	
Solvent	282	
Protein residues	402	
RMS bonds (Å)	0.002	
RMS angles (°)	0.45	
Ramachandran favored (%)	98.99	
Ramachandran allowed (%)	1.01	
Ramachandran outliers (%)	0.00	
Rotamer outliers (%)	0.95	
Average B-factor (Å^2^)	43.6	
Macromolecules	43.2	
Solvent	47.6	
PDB accession number	5OVY	


*The values in parenthesis are for the last resolution shell.*

### Docking of TAC Analogs Into the Ligand Binding Site

Docking studies was performed with *PyRx* (Dallakyan and Olson, 2015) running *AutoDock Vina* (Trott and Olson, 2010). Search was done with grid dimensions of 39.45, 39.05, 29.25 Å and origin coordinates at *x* = -17.8, *y* = 6.9, *z* = 0.63. The search was performed on chain B of the crystal structure of MAB_4384 without any additional model modification.

## Results

### MAB_4384 Specifically Regulates Susceptibility to TAC Analogs in *M. abscessus*

**FIGURE 1:**
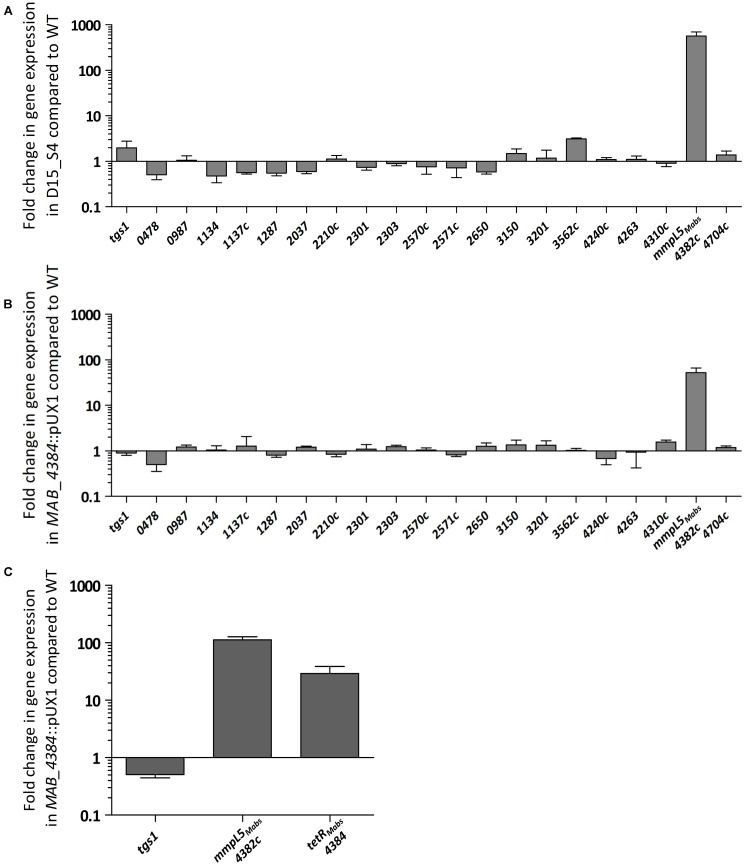
MAB_4384 is a specific repressor of the *mmpS5/mmpL5* locus in *M. abscessus*. Transcriptional profile of 19 *mmpL* in *M. abscessus* expressed in fold induction relative to expression in the wild-type strain (CIP104536^T^) in **(A)** the D15_S4 spontaneous mutant resistant to TAC analogs containing a stop codon in *MAB_4384* and in **(B)** *MAB_4384*::pUX1 in which *MAB_4384* has been disrupted by homologous recombination. *tgs1* was included as a non-relevant control gene. Error bars indicate standard deviation. **(C)** Expression of MAB_*4384* in *M. abscessus.* Fold induction levels of *MAB_4384* were calculated in *MAB_4384*::pUX1 relative to the parental strain. Error bars indicate standard deviation. Relative gene expression was calculated using the ΔΔCt method with correction for PCR efficiency. Data is representative of three independent experiments.

We recently showed that mutations in the MAB_4384 regulator were associated with the transcriptional induction of the divergently oriented adjacent genes coding for an MmpS5/MmpL5 efflux pump and accounting for high resistance levels toward various TAC analogs (Halloum et al., 2017). To gain more insight into this drug resistance mechanism in *M. abscessus*, detailed genetic, functional and structural characterizations of the MAB_4384 regulator were undertaken. First, the expression profile of 19 *mmpL* genes was analyzed by qRT-PCR using the *M. abscessus* D15_S4 strain which possesses an early stop codon in *MAB_4384* resulting in high resistance levels to the TAC analogs D6, D15 and D17 (MIC > 200 μg/mL), presumably due to derepression of the MmpS5/MmpL5 efflux pump machinery (Halloum et al., 2017). The results clearly showed a pronounced increase in the expression level of *MAB_4382c* (*mmpL5*) mRNA in D15_S4 in comparison to the wild-type strain as reported previously, while no marked effect on the remaining *mmpL* genes was detected (**Figure 1A**). The expression levels of *tgs1*, encoding the primary triacylglycerol synthase responsible for the accumulation of triglycerides in *M. abscessus* (Viljoen et al., 2016) was included as unrelated gene control. As expected, expression of *tgs1* stayed unchanged (**Figure 1A**). To further confirm these results, the *MAB_4384* and *MAB_4382c* genes were inactivated by homologous recombination using the recently developed genetic tool dedicated to facilitate gene disruption in *M. abscessus* (Viljoen et al., 2018), as illustrated in Supplementary Figures S1A,B. The mutant strain, designated *MAB_4384*::pUX1, failed to show morphological changes (Supplementary Figure S1C) and grew similarly to its parental strain and the *MAB_4382c*::pUX1 mutant (Supplementary Figure S1D), suggesting that *MAB_4384* does not play a significant role under normal *in vitro* conditions. However, this mutant exhibited high resistance levels to D6, D15, and D17 (MIC > 200 μg/mL, corresponding to >8-, >32-, and >16-fold-increases in MIC levels, respectively) (**Table 2**) similarly to our previous results for D15_S4 (Halloum et al., 2017), thus validating the expected phenotype of the strain. Analysis of the transcriptional profile of all 19 *mmpL* genes in *MAB_4384*::pUX1 confirmed the results obtained in the D15_S4 strain (**Figure 1B**). Interestingly, expression of *MAB_4384* itself was significantly induced in *MAB_4384*::pUX1, (**Figure 1C**), albeit lower than the expression level of *mmpL5*, thus indicating that MAB_4384 is self-regulated.

**Table 2:** Drug susceptibility profile of *M. abscessus* S strains inactivated in either *MAB_4384* (*tetR* gene) or *MAB_4382c* (*mmpL5* gene).

		MIC (μg/mL)				
		
Strain		D6	D15	D17	CFZ	BDQ
CIP104536^T^		25	6.2	12.5	1.6	0.05
*MAB_4384*::pUX1		>200	>200	>200	1.6	0.05
*MAB_4382c*::pUX1		12.5	6.2	6.2	1.6	0.05


*The MIC (μg/mL) was determined in CaMH medium. Data are representative of three independent experiments. CFZ, clofazimine; BDQ, bedaquiline.*

Overall, these results suggest that MAB_4384 is a unique and highly specific regulator controlling expression of *mmpL5* in *M. abscessus*, which was strongly up-regulated in the absence of MAB_4384.

### MAB_4384 Negatively Regulates Expression of *mmpS5/mmpL5*

**FIGURE 2:**
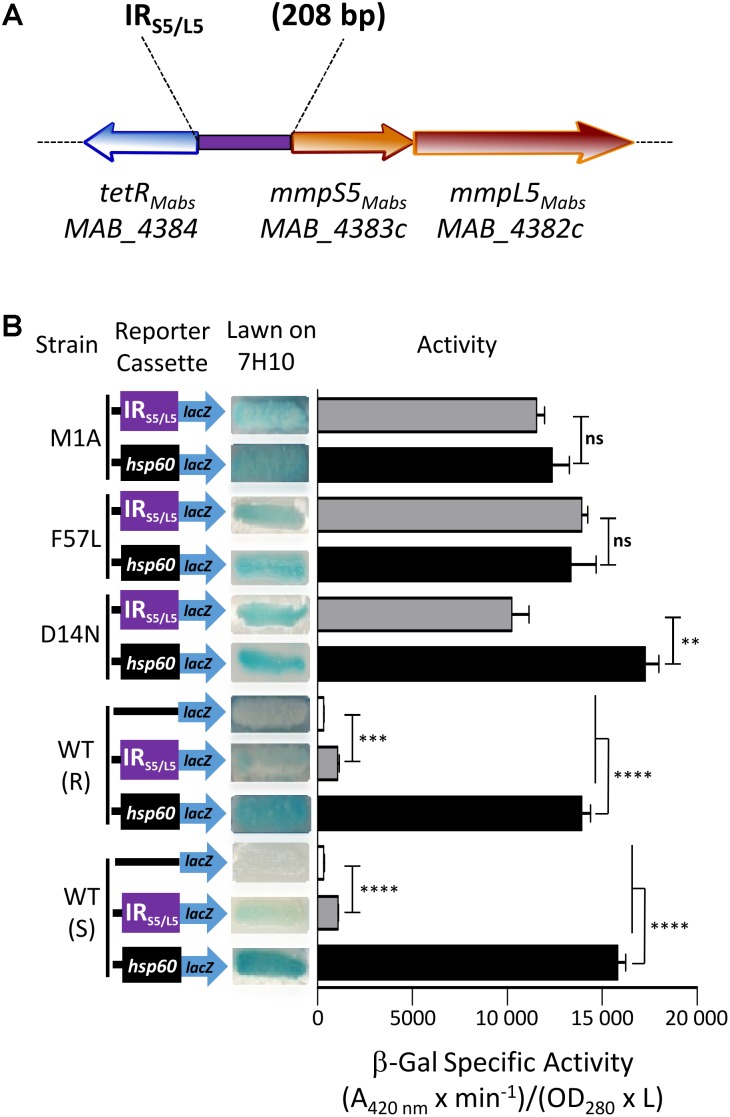
Identification of a MAB_4384-dependent regulatory promoter region in *M. abscessus*. **(A)** Schematic representation of the genome organization of *MAB_4384* and the *mmpS5/mmpL5* gene cluster in *M. abscessus* with the purple rectangle indicating the IR_S5/L5_ intergenic region cloned upstream of the *lacZ* reporter construct. **(B)** The effect of MAB_4384 on *mmpS5/mmpL5* expression was assayed by constructing a plasmid with the *lacZ* reporter under control of IR_S5/L5_. A positive control plasmid consisting of the constitutive expression of *lacZ* under the control of the *hsp60* promoter and a negative control consisting of a promoter-less *lacZ* were also generated. All constructs were introduced into wild-type smooth (S) and rough (R) variants as well as into three different strains harboring single point mutations in MAB_4384 (M1A, D14N and F57L replacements). Exponentially growing *M. abscessus* cultures were streaked onto 7H10 plates containing 100 μg/mL kanamycin and 50 μg/mL X-gal. The plates were subsequently incubated for 3–4 days at 37°C and visualized for their appearance with respect to white-to-blue coloration. The β-galactosidase specific activity (SA_β-Gal_) was quantified in liquid cultures using ONPG as a substrate. Results were obtained from three independent experiments and the error bars represent standard deviation. The capped lines indicate the groups compared. For statistical analysis, the Student’s *t*-test was applied with ns, ^∗∗^, ^∗∗∗^, ^∗∗∗∗^ indicating non-significant, *p* < 0.01, *p* < 0.001, and *p* < 0.0001, respectively.

The pMV261_P_S5/L5__*lacZ* plasmid was constructed, containing the β-galactosidase gene as a reporter in *M. abscessus* to further confirm the negative regulation of MAB_4384 on the target gene (*mmpS5/mmL5* locus) expression. To do this, the 208 bp intergenic region located between *MAB_4384* and *mmpS5/mmpL5* (**Figure 2A**), designated IR_S5/L5_, was cloned upstream of *lacZ*. pMV261_P_S5/L5__*lacZ* was subsequently introduced in parental smooth (S) and rough (R) variants of *M. abscessus* as well as in three different strains carrying single point mutations in MAB_4384 (M1A, F57L, and D14N), previously selected for their high resistance phenotype to TAC analogs (Halloum et al., 2017). In addition, pMV261_P_*hsp*60__*lacZ* allowing constitutive expression of *lacZ* under the control of the strong *hsp60* promoter was produced. As expected, pMV261_P_*hsp*60__*lacZ* led to high expression of *lacZ* in the wild-type strain and in the mutants compared to the promoter-less plasmid, as evidenced by the production of intense blue colonies and a strong β-Gal activity (**Figure 2B**). In contrast, whereas IR_S5/L5_ resulted in very low expression of LacZ in the wild-type strains, characterized by a pale blue color on plates and low β-Gal activity in liquid-grown cultures, a pronounced *lacZ* induction was detected in all three mutant strains (**Figure 2B**). Strikingly, the *lacZ* expression levels in these strains was almost comparable to the one observed in the pMV261_P_*hsp*60__*lacZ*-containing strains.

Overall, these results indicate that expression of *lacZ* is strongly repressed in the presence of an intact MAB_4384 regulator and that under derepressed conditions, the promoter driving expression of *mmpS5/mmpL5* appears almost as strong as the *hsp60* promoter.

### MAB_4384 Binds to a Palindromic Sequence Within IR_S5/L5_

**FIGURE 3:**
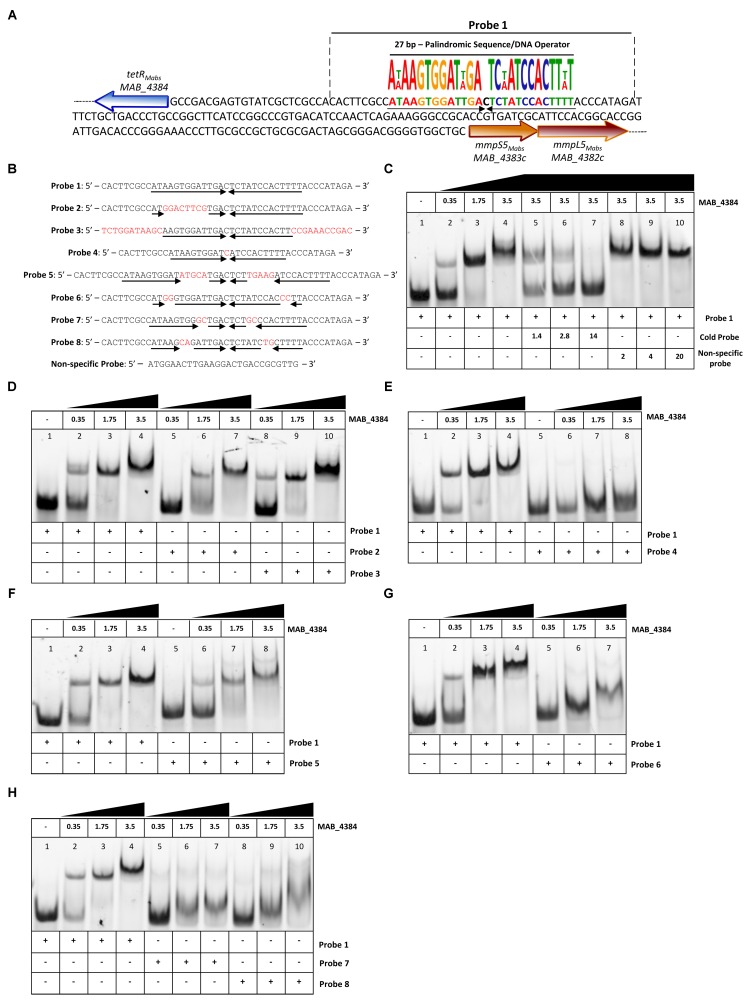
Binding activity of MAB_4384 to a palindromic region within IR_S5/L5_. **(A)** DNA sequence of IR_S5/L5_ and representation of the operator composed of a 27 bp region containing two degenerated inverted sequences of 13 nucleotides each (black arrows) and separated by a one nucleotide spacer. The probe used to perform the EMSA is delimited by dotted lines (Probe 1). **(B)** DNA sequences of all the various 5′ fluorescein-labeled probes used in this study. **(C)** EMSA and competition assay using probe 1. Protein and DNA concentrations are expressed in μM. In competition assays, the concentration of Probe 1 was fixed at 280 nM. Gel shifts were revealed by fluorescence emission. **(D–H)** EMSA using Probes 2 to 8, each time compared to the shift profile obtained with Probe 1. Experiments were reproduced three times with similar results.

Motif-based sequence analysis using MEME (Bailey et al., 2009) revealed the presence of a 27 bp segment within the divergently oriented IR_S5/L5_ intergenic region and harboring a palindromic sequence (**Figure 3A**). To test whether this motif represents a DNA binding site for MAB_4384, EMSA was first performed using increasing concentrations of purified MAB_4384 expressed in *E. coli* in the presence of a 45 bp fragment of IR_S5/L5_ (Probe 1; **Figure 3B**) carrying extra nucleotides flanking the palindromic sequence. Under these conditions, a DNA–protein complex was seen (**Figure 3C**). To confirm the specificity of the binding, a competition assay with increasing concentrations of the corresponding unlabeled probe (cold probe) was carried out, leading to a dose-dependent decrease of the DNA–protein complex (**Figure 3C**). In addition, in the presence of an excess of a non-related labeled probe, the shift was maintained, thus indicating that a specific protein–DNA complex was seen only when MAB_4384 was incubated with DNA containing the specific inverted repeat sequence. To better define the minimal motif and the importance of the nucleotides involved in recognition and binding of the protein, a large set of fluorescein-labeled probes differing in their size and/or sequence were next assayed (**Figure 3B**). In the presence of Probe 2, in which only the right inverted sequence was conserved, a delay in the DNA shift was observed (partial in the presence of 1.75 μM of protein as compared to the reaction in the presence of Probe 1). Shifts using Probe 3, where the extra nucleotides surrounding the palindromic sequence were changed randomly were comparable to those obtained with Probe 1 (**Figure 3D**), indicating that the extra-palindromic sequence does not influence protein binding. With Probe 4, where the inverted repeats were shortened by six nucleotides, the formation of the DNA–protein complex was severely impeded even with the highest concentration of protein tested (3.5 μM) (**Figure 3E**). Increasing the spacer between the two inverted repeats by 10 nucleotides (Probe 5) negatively impacted the shift (**Figure 3F**). Substitutions of two nucleotides at the extremities in each repeat sequence (Probe 6) was accompanied by a pronounced shift alteration (**Figure 3G**) and similar results were obtained when di-nucleotide substitutions occurred at other positions within the conserved palindromic sequence (Probes 7 and 8) (**Figure 3H**).

Overall, these results confirm that a strict preservation of this inverted sequence and space separating these two motifs are crucial for binding of MAB_4384 to its target.

### Asp14 and Phe57 Are Critical for Optimal DNA-Binding Activity of MAB_4384

**FIGURE 4:**
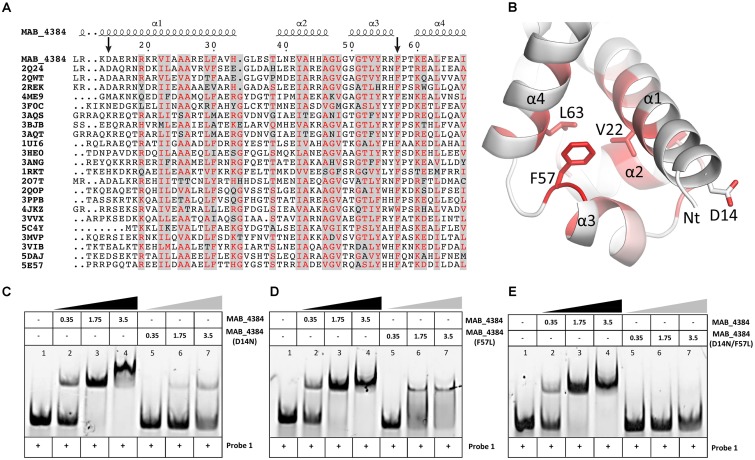
D14N and F57L mutations abrogate binding of MAB_4384 to its palindromic DNA target. **(A)** Multiple primary sequence alignments of MAB_4384 N-terminus with other TetR family members whose crystal structures are known (PDB code indicated) from different microorganisms showing the conservation of the Asp14 and Phe57 residues (indicated with black arrows). **(B)** Mapping of the mutations on the crystal structure of MAB_4384, the degree of residue conservation (obtained from the sequence alignment in **A**) is represented by the coloration, from white (low conservation) to red (high conservation). **(A,B)** Were prepared using the ENDscript server (http://endscript.ibcp.fr/ESPript/ENDscript/). EMSA were performed using increasing concentrations (in μM) of the purified MAB_4384 (D14N) **(C)**, MAB_4384 (F57L) **(D)** or MAB_4384 (D14N/F57L) **(E)** in the presence of Probe 1. Wild-type MAB_4384 was included as a positive control in each assay. The concentration of Probe 1 was fixed at 280 nM. Gel shifts were revealed by fluorescence emission. Three independent experiments were performed with similar results.

Multiple primary sequence alignments of the MAB_4384 N-terminus with other TetR regulators with known three-dimensional structures indicate that the N-terminus Asp14 residue is well conserved in several other Tet regulators (**Figure 4A**). Similarly, Phe57 was also found to be part of a highly conserved stretch of amino acids in these proteins, although, in some instances, Phe was replaced by bulky/hydrophobic residues (**Figure 4A**). The importance of the conservation of these two residues for the function of MAB_4384 and presumably also for that of the other TetR regulators, was next assessed by EMSA using the purified MAB_4384 mutated variants. As compared to the shift profile with wild-type MAB_4384, the production of the DNA–protein complex was severely impaired in the presence of either MAB_4384 (D14N) (**Figure 4C**) or MAB_4384 (F57L) (**Figure 4D**) and fully abrogated in the presence of the double mutant (D14N/F57L) (**Figure 4E**).

Overall, these results support the importance of both residues in the DNA-binding capacity of MAB_4384 and the impaired ability of the mutants to bind to the operator is in agreement with the derepression of *lacZ* transcription in the *M. abscessus* strains carrying the D14N or F57L mutations (**Figure 2B**).

### Oligomeric States of MAB_4384 and MAB_4384:DNA in Solution

**FIGURE 5:**
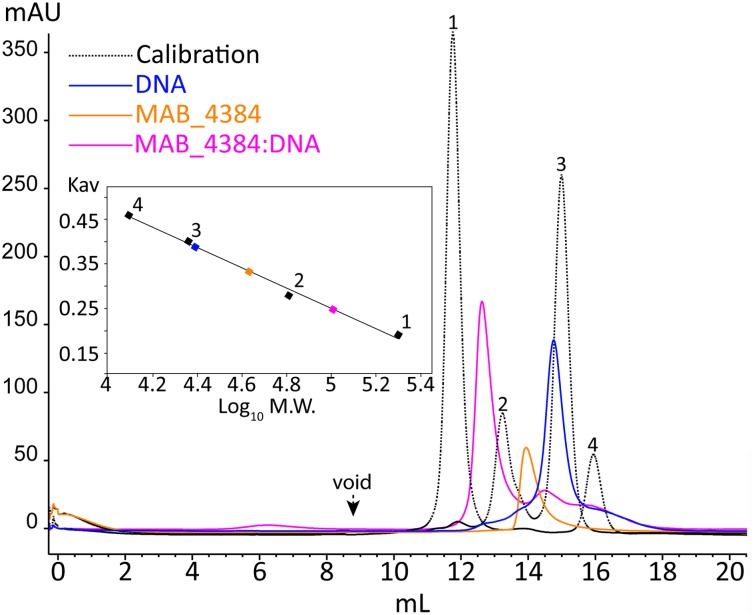
Oligomerization of MAB_4384 and the MAB_4384:DNA complex in solution. The oligomeric states of MAB_4384 alone or complexed to its DNA target were determined by size exclusion chromatography. The elution profile of the proteins, used for the calibration is displayed as a dashed black line. Calibration was established using β-amylase (200 kDa) (1), bovine serum albumin (66 kDa) (2), carbonic anhydrase (29 kDa) (3), and cytochrome C (12.4 kDa) (4), eluting with estimated volumes of 11.7, 13.2, 14.9, 15.9 mL, respectively. The void volume was determined with the elution volume (8.8 mL) of dextran blue. MAB_4384 (dimer) was at a concentration of 3.9 mg/mL. The elution profiles of MAB_4384 dimer, DNA target and MAB_4384 bound to the DNA target are shown in orange, blue, and pink and their respective elution peaks were at 13.9, 14.7, and 12.6 mL. *K*_av_ indicates the partition coefficient.

To further characterize the MAB_4384:DNA complex formation, we next assessed its stability in solution by SEC (**Figure 5**). The oligomeric state of MAB_4384 in solution has an apparent 42.6 kDa molecular weight as compared to its 24.7 kDa theoretical molecular mass calculated from its primary sequence, thus highlighting the dimeric state of MAB_4384 in solution. MAB_4384 (dimer) was next incubated with the non-fluorescent DNA Probe 1 (**Figure 3A**) in a 1:1 molar ratio. A stable complex elution peak at 12.6 mL clearly shifted from the elution peak of MAB_4384 and DNA alone (**Figure 5**), allowing deduction of the molecular mass of the protein:DNA complex at 102 kDa. As the DNA alone in solution appeared on SEC as a 24.5 kDa molecule, these results strongly suggest the existence of two MAB_4384 dimers bound to one DNA molecule as such a complex would possess a molecular mass of 109.7 kDa (2 × 42.6 kDa + 24.5 kDa). This 2-to-1 binding mode was further corroborated by the fact that an elution peak corresponding to free DNA can be seen at 14.4 mL when we mixed the MAB_4384 dimer and DNA in a 1:1 ratio. Moreover, increasing the molar ratio of the MAB_4384 dimer:DNA complex (2:1 and 3:1) did not yield larger protein:DNA complexes (data not shown), suggesting that, at a 1:1 molar ratio, the operator is already saturated by the protein. This observation is not unique as two TetR dimers have been shown to bind their DNA targets in other microorganisms, such as in *Staphylococcus aureus* (Grkovic et al., 2001) or in *Thermus thermophilus* (Agari et al., 2012).

### Crystal Structure of MAB_4384

To understand, at a molecular basis, how the D14N or F57L mutations generate resistance to TAC analogs, we first crystallized and determined the X-ray structure of MAB_4384. Although the structure of the protein could not be solved by molecular replacement, the phase problem was overcome with the SAD method using crystals of selenomethionine-substituted MAB_4384 (**Table 1**). The crystal structure of the native protein was subsequently solved with the partial model obtained from the SAD data and refined to a resolution of 1.9 Å. The asymmetric unit contains two subunits. Chain A was modeled from residues Asp14 to Thr213, indicating that the first thirteen residues, one residual Gly residue from the tag and the last eight residues in the C-terminus were not visible in the electron density. Chain B showed also disordered regions as the first ten residues, one Gly residue from the tag in N-terminus as well as the last nine residues in the C-terminus, could not be modeled. Analysis of the crystal packing using the *PISA* server (Krissinel and Henrick, 2007) predicted the existence of a stable homodimer formed within the crystal, consistent with other TetR regulators (Cuthbertson and Nodwell, 2013) and with the SEC profile of MAB_4384 in solution (**Figure 5**).

**FIGURE 6:**
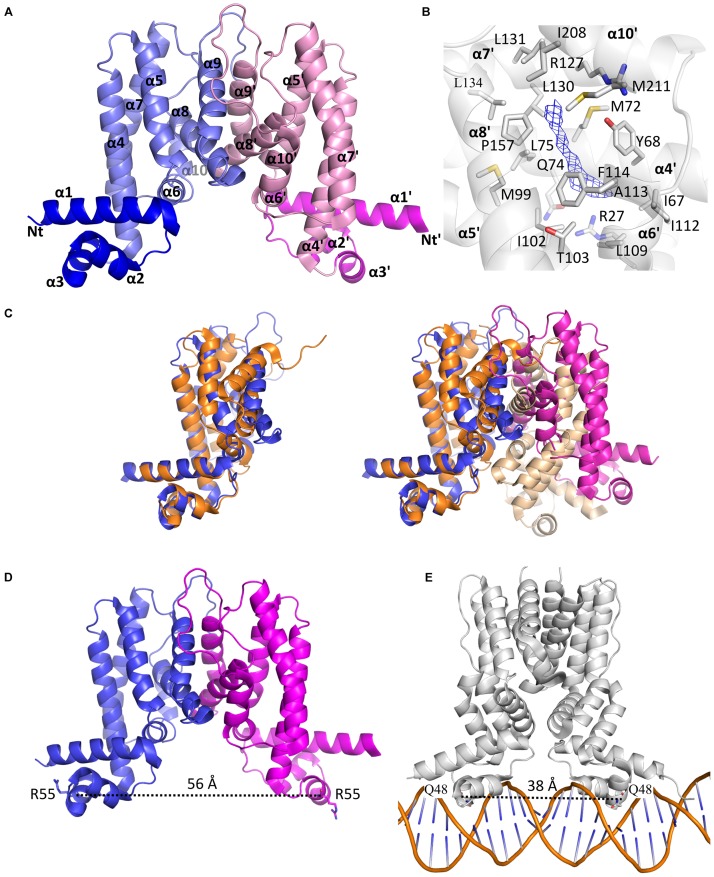
The homodimeric crystal structure of MAB_4384. **(A)** Overall structure of the MAB_4384 dimer displayed as cartoon representation. The LBDs of each subunit are colored in slate and pink while the DNA binding domains are colored in blue and magenta. Helices are indicated by the α signs followed by numbers, Nt and Ct stands for N-terminus and C-terminus and ’ is for chain B. **(B)** Putative ligand binding pocket in the LBD of MAB_4384. The Fo-Fc simulated annealed omit map contoured at 3 σ level is shown in blue. Residues that are 4 Å around the electron density blob and that are potential amino acids of the ligand binding site are shown as sticks. **(C)** Structural comparison of MAB_4384 with the crystal structure of the *M. smegmatis* LfrR repressor (PDB id: 2V57). The left panel represents the superposition of one monomer of MAB_4384 (in blue) on one monomer of LfrR (in orange). The superposition of the two homodimers is shown on the right panel, the two subunits of MAB_4384 are in blue and magenta and the two monomers of LfrR are in orange and wheat. **(D,E)** The figures compare the distance between the two DNA binding domains in MAB_4384 **(D)** and in the crystal structure of the *M. smegmatis* TetR Ms6564 protein bound to its DNA target (PDB id: 4JL3).

The two subunits are very similar as their superposition leads to a root mean square deviation (r.m.s.d.) of 0.53 Å over 198 aligned residues. The N-terminus comprises the DNA binding domain (DBD), followed by the LBD. The DBD is composed of three α-helices α1: residues 12–33, α2: 39–46, and α3: 50–56, where helices 2 and 3 form a helix-turn-helix (HTH) motif. The LBD is made of seven α-helices, α4: 60–82, α5: 88–104, α6: 107–114, α7: 121–143, α8: 155–170, α9: 178–188, and α10: 205–211 (**Figure 6A**). The surface of dimerization of about 1,700 Å^2^ is mediated by 31 residues mainly from helices α8 and α9 of each subunit and involves numerous interactions notably five salt bridges, fourteen hydrogen bonds and van der Waals interactions.

Interestingly, we noticed the presence of extra electron density in the LBD of chain B in a rather hydrophobic pocket (**Figure 6B**). Although we could not interpret this density, we hypothesize that it may correspond to a compound present in the crystallization solution, such as PEG. Search for structural homologs in the PDBeFold server indicated that the closest structure to MAB_4384 corresponds to the LfrR TetR transcriptional regulator from *Mycobacterium smegmatis* bound to proflavin (PDB id : 2V57) (Bellinzoni et al., 2009) with an r.m.s.d. of 2.6 Å and sharing 16% primary sequence identity with MAB_4384. However, only one subunit of each structure could be superposed as the overall dimers differed largely (**Figure 6C**). LrfR represses the expression of the LfrA efflux pump (Buroni et al., 2006) and mediates resistance to ethidium bromide, acriflavine, and fluoroquinolones (Takiff et al., 1996).

Due to the occurrence of an extra electron density within the LBD of MAB_4384 and that the closest structure of MAB_4384 is LfrR in its ligand bound form, it is very likely that MAB_4384 was crystallized in its open conformation, i.e., its derepressed form that is not able to interact with DNA. This was assessed by determining the distance between two residues from the DBD susceptible to interact with DNA. Residues Arg55 from chains A and B are about 56 Å apart (**Figure 6D**). In comparison, the distances between the equivalent residues in various TetR:DNA complexes are largely reduced. In the TetR:DNA complex (PDB id: 4PXI) from *Streptomyces coelicolor* this distance is 45 Å (Bhukya et al., 2014), in the TetR:DNA complex from *M. smegmatis* (PDB id: 4JL3) (Yang et al., 2013) (**Figure 6E**), *E. coli* (PDB id: 1QPI) (Orth et al., 2000), *Corynebacterium glutamicum* (PDB id: 2YVH) (Itou et al., 2010) or *Staphylococcus aureus* (PDB id: 1JT0) (Schumacher et al., 2002) the distances are 38 Å, 30 Å, 42 Å, and 37 Å, respectively. From these results it can be inferred that the DBDs of MAB_4384 are too far from each other to bind to the DNA groove. These observations combined with the presence of an unidentified ligand in the LBD strongly suggest that the MAB_4384 structure is in an open conformation.

### Structural Basis of the Resistant Phenotype of the Mutants

To determine the impact of the mutations in the spontaneous resistant *M. abscessus* mutants, the D14N and F57L residues were mapped on the crystal structure of MAB_4384. Asp14 is located at the beginning of helix α1 and is conserved in several TetR protein members (**Figures 4A,B**). Residues from helix α1 are often found in contact with DNA as seen in several TetR:DNA crystal structures. Nonetheless, due to the acidic nature of Asp, it is more likely that this residue repulses DNA. We, therefore, hypothesize that it may instead contribute to the correct positioning of other residues located in its close vicinity. Alternatively, repulsion may promote important interactions of DNA with other residues. Indeed, in other collected datasets at lower resolution (data not shown), Asp14 was found to establish a salt bridge interaction, thereby stabilizing the side chain of Arg17 that could interact with DNA. In the absence of a crystal structure of MAB_4384 bound to DNA it is, however, difficult to convincingly affirm the impact of the D14N substitution. However, neither the repulsion of DNA nor the establishment of a salt bridge would be possible if Asn is present instead of Asp, presumably explaining the loss of DNA binding activity of the TetR D14N mutant.

The role of Phe57 situated on helix α3 is more obvious as this position appears always occupied by bulky residues (Phe, Tyr, or Trp) in numerous TetR proteins (**Figure 4A**). The side chain of Phe57 contacts the side chains of Val22 from helix α1 and Leu63 from helix α4 (**Figure 4B**). Phe57 is very likely to perform an important structural role in stabilizing the DBD. Replacement with a less bulky side chain such as Leu would abolish these contacts with helices α1 and α4 residues, thus perturbing the overall structural fold of this domain and suppressing the DNA-binding capacity of MAB_4384.

### Drug Recognition of MAB_4384 Induces Expression of MmpS5/MmpL5

**FIGURE 7:**
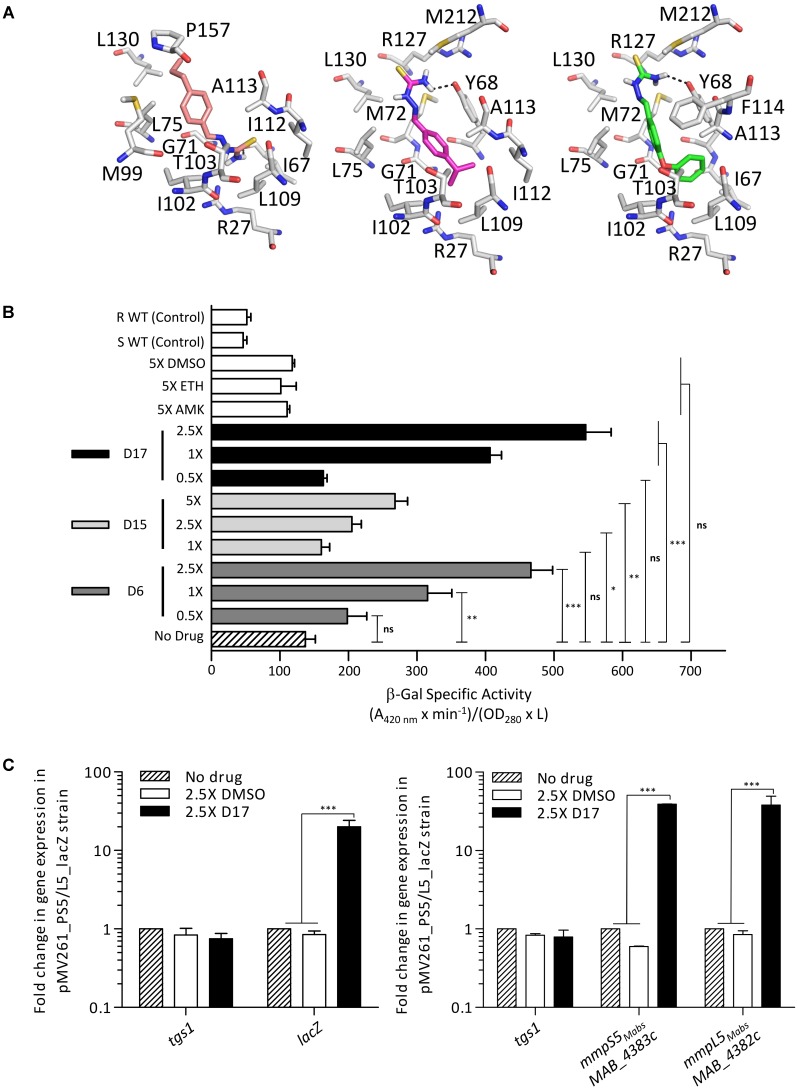
IR_S5/L5_ can be induced by structural analogs of thiacetazone. **(A)** Docking of TAC derivatives in the ligand binding site of MAB_4384. All the residues involved in van der Waals, hydrophobic bonds or hydrogen bonds (in black dashes) are displayed as sticks. D15 in salmon has a binding energy of ΔG = –6.6 kcal/mol, D6 in magenta has a ΔG = –7.3 kcal/mol, and D17 seems to bind slightly stronger with a ΔG = –8.3kcal/mol. **(B)** Conditional induction of *lacZ* by structural analogs of TAC in *M. abscessus*. Induction of β-Gal activity in wild-type *M. abscessus* S carrying pMV261_P_S5/L5__*lacZ* was assayed using mid-log phase cultures incubated with increasing drug concentrations varying from 1X to 5X the MIC for D15 and varying from 0.5X to 2.5X the MIC for D6 and D17. Inductions were performed for 96 h at 37°C. The β-galactosidase specific activity (SA_β-Gal_) was quantified in liquid cultures using ONPG as a substrate. Amikacin (AMK) and ethionamide (ETH) were included as unrelated drug controls. **(C)** Transcriptional profile of *lacZ* in the *M. abscessus* pMV261_P_S5/L5__*lacZ* reporter strain exposed to 2.5X the MIC of D17 for 8 h (left). *tgs1* was included as a non-relevant control. Replacing D17 by an equal volume of DMSO had no effect on *lacZ* transcription. Transcriptional induction of *mmpS5_Mabs_* and *mmpL5_Mabs_* following exposure to 2.5X the MIC of D17 for 8 h (right). Results were obtained from three independent experiments and the error bars represent standard deviation. For statistical analysis the Student’s *t*-test was applied with ns, ^∗^, ^∗∗^, ^∗∗∗^, ^∗∗∗∗^ indicating non-significant, *p* < 0.05, *p* < 0.01, *p* < 0.001, and *p* < 0.0001, respectively.

TetR regulators can respond to small molecules and the best characterized member of this family of regulators is *E. coli* TetR itself. It confers resistance to tetracycline by regulating the expression of the tetracycline TetA efflux pump (Hillen and Berens, 1994). When tetracycline binds to TetR, the regulator loses affinity for the operators, conducting derepression of *tetA* and extrusion of tetracycline out of the bacteria (Lederer et al., 1995). To investigate whether TAC derivatives could bind to the LBD of MAB_4384, *in silico* docking was performed. Despite using a large grid box covering the entire LBD, all three compounds seem to be accommodated by the same binding pocket (**Figure 7A**). Interestingly, this pocket positioned exactly where the extra electron density was seen in the LBD (**Figure 6B**). All the compounds bind with similar energies in the aforementioned hydrophobic binding pocket. A slightly stronger interaction for the most hydrophobic derivative D17 was nonetheless observed. D17 and D6 that seem to bind stronger are more hydrophobic and in their best docking poses their thiosemicarbazide group is differently oriented as compared to D15.

Next, we determined whether expression of *mmpS5/mmpL5* can be conditionally induced by the substrates that are extruded by the efflux pump system. This was achieved by determining the effect of the D6, D15, and D17 analogs on LacZ production using the pMV261_P_S5/L5__*lacZ* reporter strain in *M. abscessus* incubated in Sauton’s medium with various drug concentrations consisting of 1X, 2.5X, and 5X the MIC for D15 and of 0.5X, 1X, and 2.5X for D6 and D17. Kinetic studies indicated that optimal expression was obtained after 96 h of treatment (data not shown). The LacZ assay showed that transcription was induced by the TAC analogs in a dose-dependent manner whereas non-related drugs such as amikacin or the DMSO control had no effects (**Figure 7B**). In comparison with the basal transcriptional level in Sauton’s medium (no drug control), the addition of TAC derivatives in the cultures resulted in a reproducible 2.5- to 5-fold increase in the detection of β-Gal activity with D17 being the most potent inducer at 2.5X MIC. However, ethionamide, an antitubercular drug that, like the TAC and TAC analogs, requires to be activated by the EthA monooxygenase (Baulard et al., 2000; DeBarber et al., 2000; Dover et al., 2007; Halloum et al., 2017) failed to induce *lacZ* at 5X MIC (previously determined at 16 μg/ml). Induction of *lacZ* by D17 treatment was further confirmed at a transcriptional level from the pMV261_P_*S5/L5*__*lacZ* cultures treated with 2.5× MIC of D17 for 8 h (**Figure 7C**, left). This effect was specific to D17 as no gene induction was observed in the DMSO-treated cultures. Consistently, transcription profiling of *mmpS5* and *mmpL5* in the D17-exposed cultures clearly showed a marked induction level as compared to the DMSO-treated cultures and no effect on *tgs1* expression (**Figure 7C**, right).

Together, these results support the view that TAC analogs, which are substrates of MmpS5/mmpL5, are also effectors of MAB_4384-induced transcription of *mmpS5/mmpL5.*

## Discussion

Herein, a combination of genetic, biochemical and structural studies was used to demonstrate that MAB_4384 is part of the TetR family of regulators, which represses the transcriptional expression of the MmpS5/MmpL5 efflux pump. MAB_4384 belongs to the type I class TetR family of regulators, characterized by a divergent orientation to one of the adjacent target genes (Cuthbertson and Nodwell, 2013). In *M. tuberculosis*, MmpS5/MmpL5 is under the control of the MarR repressor Rv0678 (Radhakrishnan et al., 2014) and mutations in this regulator leads to drug resistance (Andries et al., 2014; Hartkoorn et al., 2014; Zhang et al., 2015). EMSA indicated a direct binding of Rv0678 to the intergenic region located between *mmpS5* and *Rv0678*. However, shifts were also found using the promoter regions of *mmpS2-mmpL2*, *mmpS4-mmpL4*, and *Rv0991-Rv0992* (Radhakrishnan et al., 2014), suggesting that a single regulator can control expression of several *mmpS/mmpL* loci. Our analysis indicates that, despite the fact that *M. abscessus* possesses the highest number of *mmpL* genes among all mycobacterial species studied (Viljoen et al., 2017), the MAB_4384 regulator is highly specific to the *mmpS5*/*mmpL5* pair as demonstrated by the lack of transcriptional regulation of a large set of *mmpL* genes and the presence of a unique inverted DNA sequence target that was not found elsewhere in the chromosome. This unique trait might also be reflected by the modest structural homology of MAB_4384 with other TetR crystal structures. The tight regulation and the high specificity of interaction with its target DNA, however, cannot be solely explained on the basis of the MAB_4384 crystal structure and the structure of the MAB_4384:DNA complex is, therefore, greatly warranted to dissect these underlying mechanisms. Nevertheless, our structural analysis underscores the strategy employed by *M. abscessus* to acquire mutations impacting the DNA-binding capacity or the folding/stability of the DBD of MAB_4384 to become resistant.

**FIGURE 8:**
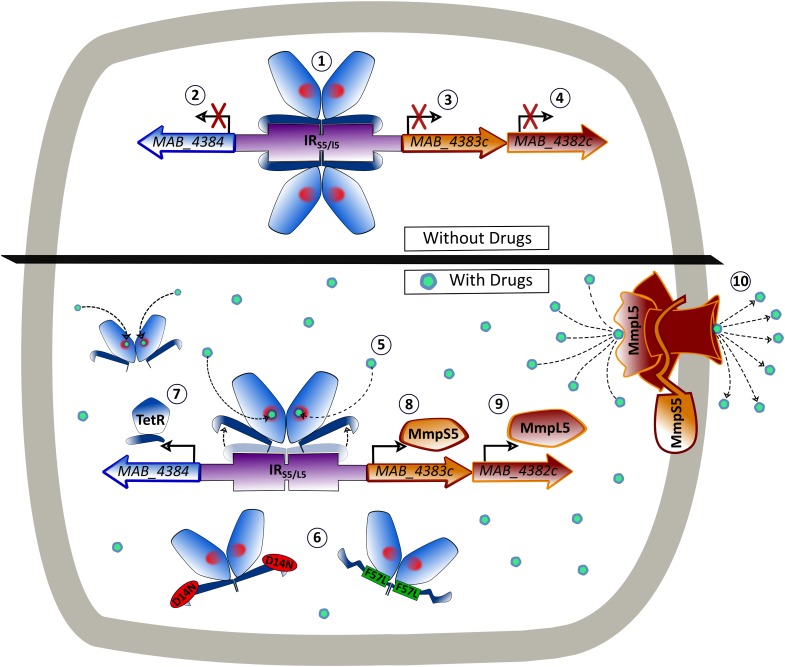
Model of the binding of MAB_4384 to its operator and regulation of the MmpS5/MmpL5 efflux pump machinery. In the absence of drug, two MAB_4384 dimers bind to their DNA operator located in the intergenic region (IR_S5/L5_) between the divergently transcribed *MAB_4384* (encoding the TetR regulator) and *MAB_4383c*/*MAB_4382c* (encoding the MmpS5/MmpL5 efflux pump) (1). This action represses the transcription of *MAB_4384* (2), *MAB_4383c* (3) and *MAB_4382c* (4), predisposing the bacteria to drug susceptibility. When the TAC derivatives bind to MAB_4384 (5) or if MAB_4384 harbors the D14N or F57L mutations (6), the regulator loses affinity for the operator, leading to derepression of *MAB_4384* itself (7), *MAB_4383c* (8) and *MAB_4382c* (9). This triggers high levels of expression of the MmpS5/MmpL5 complex in the plasma membrane and the subsequent export of the TAC analogs outside the bacteria (10), reducing susceptibility to the compounds.

EMSA and *lacZ* reporter fusions confirmed that D14N and F57L mutations, alleviating the DNA-binding activity of MAB_4384, cause a strong up-regulation of *mmpS5/mmpL5* gene expression, in agreement with our previous qRT-PCR analyses (Halloum et al., 2017). This leads to extrusion of the TAC derivatives out of the cells, contributing to the high MIC values for TAC derivatives against these mutants, as illustrated in **Figure 8**. Expression of multi-drug resistant efflux pumps can also be conditionally induced using structurally diverse substrates (Kaatz and Seo, 1995; Rosenberg et al., 2003; Buroni et al., 2006). This induction is caused by the direct interaction of these substrates with the repressors, interfering with binding of the repressors to their target operators and resulting in increased expression of the pumps. Here, we show inducible β-galactosidase activity following treatment with D6, D15, or D17, a mechanism that is very likely to be meditated by MAB_4384. This view is reinforced by the fact that docking studies highlighted the possibility that all three analogs could be accommodated in the LBD of the protein, which perfectly coincided with the extra electron density observed. Since, the LBD are remote from the DBD, the derepression of TetR family regulators involves allosteric mechanisms that include conformational changes transmitted largely within the same subunit (Ramos et al., 2005). The interaction of ligands with the LBD captures a conformational state where the DBD is repositioned relative to the LBD in a way that the dimer is prevented from binding to its target DNA. However, definitive proof of this mechanism awaits the elucidation of the crystal structure of the D17-bound form of MAB_4384, as reported for instance with the hexadecyl octanoate-bound EthR repressor (Frénois et al., 2004) or the LfrR regulator complexed with proflavine (Bellinzoni et al., 2009). Lack of inducible *lacZ* expression in *M. abscessus* cultures exposed to amikacin, for which mutations in 16S rRNA represent a major mechanism of resistance (Prammananan et al., 1998), indicates that MmpL5-mediated efflux cannot mediate resistance toward this antibiotic in line with the lack of cross-resistance toward amikacin observed for TAC derivative-spontaneous resistant mutants (Halloum et al., 2017). The specificity of the MAB_4384-driven resistance mechanism described herein is further supported by the lack of *lacZ* induction during exposure to ETH, that similarly to TAC and TAC analogs, requires bio-transformation by EthA, whose expression is also dependent on the EthR regulator belonging to the TetR family (Baulard et al., 2000; Engohang-Ndong et al., 2004; Halloum et al., 2017). Together, these findings strongly suggest that when TAC analogs bind to MAB_4384, the regulator loses affinity for its DNA target, resulting in up-regulation of *mmpS5/mmpL5* and export of the drugs from the cells (**Figure 8**). These results also point out the selectivity of this efflux-based mechanism. Indeed, no change in the MIC of clofazimine or bedaquiline were noticed in a *MAB_4384*-disrupted strain, which appears intriguing as MmpL5 has been reported as a multi-substrate efflux pump responsible for low-level resistance to both of these drugs in *M. tuberculosis* (Hartkoorn et al., 2014). The LBD of MAB_4384 potentially can accommodate bulky molecules and might thus indicate that MAB_4384 is involved in efflux of other types of compounds in addition to TAC analogs. However, we could neither dock clofazimine nor bedaquiline in the LBD of MAB_4384 (not shown). Several reasons can be put forth to explain these species-specific variations. In *M. tuberculosis*, expression of MmpL5 is under the control of a MarR regulator rather than a TetR regulator. Alternatively, we have previously reported the occurrence in *M. abscessus* of three *mmpS5/mmpL5* paralogs (Halloum et al., 2017), thus, it remains possible that either of the two remaining genes may participate in co-resistance to these drugs in *M. abscessus.*

The highly pronounced expression of *lacZ* under derepressed conditions found in the M1A, F57L, or D14N mutant strains, almost at levels similar to those driven by the strong and constitutive *hsp60* promoter, confirmed the very high expression levels of *mmpS5* and *mmpL5* detected by qRT-PCR and probably explains the very high level of resistance of the mutants (MIC > 200 μg/mL). This contrasts also with findings where MmpL5 mediates only low-levels of resistance in *M. tuberculosis* (Andries et al., 2014; Hartkoorn et al., 2014), presumably because expression of *mmpL5* is driven by a weaker promoter than in *M. abscessus.* That *mmpS5/mmpL5* expression is tightly controlled suggests that the MmpS5/MmpL5 machinery may exert an important function in the assembly and/or maintenance of the cell wall by exporting a yet unidentified lipid, as already reported for several MmpL transporters in *M. tuberculosis* (Chalut, 2016; Viljoen et al., 2017). Alternatively, they may participate in adaptation during the infection process. However, the growth curves of the wild-type or the strain constitutively expressing high levels of MmpL5 (due to the M1A mutation in MAB_4384) were comparable *in vitro.* In addition, microinjections of the different strains were done in the zebrafish embryo, an animal model previously developed to study the early events of the *M. abscessus* infection (Bernut et al., 2014, 2015). No differences in virulence were noticed between the wild-type and MAB_4384 (M1A) strains (Supplementary Figure S2). Interestingly, the *mmpS5/mmpL5* locus was found to be induced when *M. abscessus* was exposed to a defined, synthetic medium that mimics the composition of CF sputum (Miranda-CasoLuengo et al., 2016). This may be part of a complex adaptive transcriptional response to the mucus layer of the CF airways that leads to the chronic infections of *M. abscessus*.

In summary, this study provides new functional and structural insights into TetR-dependent regulation of MmpL efflux pumps in mycobacteria. Considering the exceptionally high abundance of TetR transcriptional regulators (more than 130) as well as the important MmpL repertoire (around 30) in *M. abscessus*, one can anticipate that mechanisms similar to the one described here are exploited by this pathogen to express its intrinsic resistance level to other antibiotics.

## Ethics Statement

Zebrafish experiments were done at IRIM, according to European Union guidelines for handling of laboratory animals(http://ec.europa.eu/environment/chemicals/lab_animals/home_en.htm) and approved by the Direction Sanitaire et Vétérinaire de l’Hérault and Comité d’Ethique pour l’Expérimentation Animale de la Région Languedoc Roussillon (CEEA-LR) under the reference CEEA-LR-1145.

## Author Contributions

MR, AVG, AV, MB, and LK acquired and analyzed the data. EG, MB, and LK wrote the manuscript. LK conceived and designed the study.

## Conflict of Interest Statement

The authors declare that the research was conducted in the absence of any commercial or financial relationships that could be construed as a potential conflict of interest.

## Footnotes

1 http://meme-suite.org

2 www.pymol.org
