# Supplementary material for: Mechanistic and Structural Insights Into the Unique TetR-Dependent Regulation of a Drug Efflux Pump in Mycobacterium abscessus
Source: Front Microbiol. 2018 Apr 5;9:649. doi: 10.3389/fmicb.2018.00649 (PMC5895659; doi:10.3389/fmicb.2018.00649)
Supplement: Supplementary file 1 [file Table_1.PDF]

Supplementary Information

Mechanistic and structural insights into the unique TetR-dependent regulation of a drug efflux pump in *Mycobacterium abscessus*

Matthias Richard[1], Ana Victoria Gutierrez[1,3], Albertus Viljoen[1], Eric Ghigo[4], Mickael Blaise[1,*] and Laurent Kremer[1,2,*]

[1]Institut de Recherche en Infectiologie de Montpellier (IRIM), Université de Montpellier, CNRS UMR 9004, Montpellier, France.
[2]INSERM, IRIM, 34293 Montpellier, France.
[3]Unité de Recherche Microbes, Evolution, Phylogeny and Infection (MEPHI), Institut Hospitalier Universitaire Méditerranée-Infection, Cedex 05, 13385 Marseille, France.
[4]CNRS, Campus Joseph Aiguier, 31 Chemin Joseph Aiguier, 13009 Marseille, France.

[*]To whom correspondence should be addressed:
Tel: (+33) 4 34 35 94 47; E-mail: laurent.kremer@irim.cnrs.fr; mickael.blaise@irim.cnrs.fr

Running title: TetR-dependent regulation of MmpL expression in *M. abscessus*.

Keywords: *Mycobacterium abscessus,* TetR regulator, MmpL, efflux pump, structure, thiacetazone analogues, EMSA.

Table S1. List of bacterial strains and plasmids used in this study.

Name	Description/genotype	Marker	Reference
**Strains**			
M. abscessus S	*Mabs sensu stricto*, strain $CIP104536^T$, smooth	_	Laboratoire de Référence des Mycobactéries
M. abscessus R	*Mabs sensu stricto*, strain CIP104536 ^{T}, rough	_	Laboratoire de Référence des Mycobactéries
M. abscessus S Δ*mmpL5*	*Mabs* mutant obtained by recombination in the smooth parental strain $CIP104536^T$	Kan	This study
M. abscessus R Δ*mmpL5*	*Mabs* mutant obtained by recombination in the rough parental strain $CIP104536^T$	Kan	This study
M. abscessus S Δ*MAB_4384*	*Mabs* mutant obtained by recombination in the smooth parental strain $CIP104536^T$	Kan	This study
M. abscessus R Δ*MAB_4384*	*Mabs* mutant obtained by recombination in the rough parental strain $CIP104536^T$	Kan	This study
M. abscessus D15_S6	*Mabs* smooth strain containing a D14N point mutation in MAB_4384 and resistant to TAC analogues	_	Halloum et al., 2017
M. abscessus D15_S7	*Mabs* smooth strain containing a F57L point mutation in MAB_4384 and resistant to TAC analogues	_	Halloum et al., 2017
M. abscessus D15_R1	*Mabs* rough strain containing a M1A point mutation in MAB_4384 and resistant to TAC analogues	_	Halloum et al., 2017
E. coli HB101	*F^- mcrB mrr hsdS20(rB^- mB^-) recA13 leuB6 ara-14 proA2 lacY1 galK2 xyl-5 mtl-1 rpsL20(SmR) glnV44 λ-*	Stp	MCLAB
E. coli XL1-Blue	*recA1 endA1 gyrA96 thi-1 hsdR17 supE44 relA1 lac* [F′ *proAB lacIqZΔM15* Tn*10* (Tetr)].	Tet	Stratagene
E. coli BL21(DE3) Rosetta2	F^- *ompT* $hsdS_B$(r_B^- m_B^-) *gal dcm* (DE3) pRARE2 (Cam^R)		Novagen
**Plasmids**			
pET32a	Vector for high-level expression of proteins fused with an N-terminal thioredoxin protein and a His tag	Amp	Novagen
pET32a_*MAB_4384*	pET32a in which *MAB_4384* is cloned in fusion with the thioredoxin/His tag sequence and containing a TEV cleavage site before MAB_4384 to remove the tags fusion	Amp	This work
pET32a_MAB_*4384(D14N)*	pET32a_*MAB_4384* carrying the D14N mutation in MAB_4384	Amp	This work

pET32a_MAB_*4384(F57L)*	pET32a_*MAB_4384* carrying the F57L mutation in MAB_4384	Amp	This work
pET32a_MAB_*4384(D14N/F57L)*	pET32a_*MAB_4384* carrying the double D14N/F57L mutation in MAB_4384	Amp	This work
pUX1	Suicide construct designed for single cross-over in mycobacteria	Kan	Viljoen et al. 2017, in preparation
pUX1_*MAB_4384*	pUX1 designed to inactivate *MAB_4384* in *M. abscessus*	Kan	This study
pUX1_*mmpL5*	pUX1 designed to inactivate *mmpL5* in *M. abscessus*	Kan	This study
pMV261	Multi-copy *E. coli*/mycobacterial shuttle vector. Cloned genes are under the control of the constitutive *hsp60* promoter	Kan	(53)
pMV261_*lacZ*	pMV261 containing the promoterless *lacZ* reporter gene	Kan	(53)
pMV261_P_{hsp60}_*lacZ*	pMV261_*lacZ* carrying the *hsp60* promoter region cloned upstream of *lacZ*	Kan	(53)
pMV261_$P_{S5/L5}$_*lacZ*	pMV261_*lacZ* carrying the promoter region of *mmpS5/mmpL5* cloned upstream of *lacZ*	Kan	This study

Amp, ampicillin; Kan, kanamycin; Tet, Tetracycline; Stp, streptomycin

Table S2: Oligonucleotides used in this study. Fw and Rev stand for forward and reverse, respectively.

**Primers**	**5' to 3' sequence**
**Cloning in pET32a**	
MAB_4384_full	Fw: TCA GGG TAC C*GA GAA TCT GTA CTT CCA GGG A* GT GAA CGG CCG TGA AGT GGC GCA TC (KpnI , Tobacco Etch Virus [TEV] cleavage site in in italic)<br>Rev: GAG AAA GCT TGA GAC GCA GTG ACA GTG AGC (HindIII))
MAB_4384_DM	Fw: GTG TAC CGC CGC **C**TT CCC ACG AAA (substituted nucleotide in bold)<br>Rev: TTT CGT GGG AA**G** GCG GCG GTA CAC (substituted nucleotide in bold)
**Cloning in pMV261_*lacZ* derivatives**	
HB101_lacZ	Fw: TAT ATA GGA TCC GAT GAC CAT GAT TAC GGA TTC ACT GGC C (BamHI)<br>Rev: TAT AGC AAG CTT TAC GCG AAA TAC GGG CAG ACA TGG (HindIII)
*MAB_4384_* $P_{S5/L5}$	Fw: *TCT AGA* GCC GAC GAG TGT ATC GCT CG (XbaI)<br>Rev: *GGA TCC* CGC AGC CAC CCC GTC CCG CT (BamHI)
**Cloning in pUX1**	
*MAB_4384::*pUX1	Fw: GAG AGA GCT AGC CCT TTT CGA GGC AAT TTA CG (NheI)<br>Rev: GAG AGA GGA TCC CGG TAA GCA CTC CGA AGA AG (BamHI)<br>Conf: CGT AAC CGG AAG CGT GTA ATC
*mmpL5::*pUX1	Fw: TCAG GGA TCC GGC AAC TGC CGT CAT TAA CT<br>Rev: GAG AGC TAG CCC GGT CGT TAT AGG TGG TGT<br>Conf: CGG GTT CAT CTT GGC TTT AGA
**Sequencing**	
pMV5'	CGC CCG GCC AGC GTA AGT AGC
pMV3'	GCC TGG CAG TCG ATC GTA CG
pMV3' Ext	TTG AGA CAC AAC GTC GCT TT
NheIpUX1	ACGGCATGGACGAGCTGTAC
**qRT-PCR**	
sigA	Fw: CAC ATG GTC GAG GTC ATC AA<br>Rev: TGG ATT TCC AGC ACC TTC TC
MAB_4384	Fw: CGT AAC CGG AAG CGT GTA ATC<br>Rev: GGA AAG CGG CGG TAC AC
MAB_3551C (tgs1)	Fw: CAC CGT CTA CTA CGG AAT CAA C<br>Rev: TGC GCA GCC TCC AAT AAT
MAB_0478	Fw: CCACTCCGCAGATGATTACT<br>Rev: GCGATGGTGGCATGAAATAG
MAB_0987c	Fw: CCGCCTATCAGACGATGTATAAG<br>Rev: CGTGTGAACGACAGACAGAA
MAB_1134c	Fw: CCGGAAGGTCTTTCGCATATC<br>Rev: CGAGTTCGATCTTGGCATCTT
MAB_1137c	Fw: CTCGGGCTACCTCAATAACATC<br>Rev: GCCTTGTGGCGGTAGATAAA
MAB_1287	Fw: AACAACAGCTCCACCAACT<br>Rev: CTCCTTGACGTCCTCGATATTC

MAB_2037	Fw: GGGACAATGGAGGAGATGAAG<br>Rev: GTGCGGTTCCCAGTAGTAATAG
MAB_2210c	Fw: CCAAGCGGTACAAGGCATTA<br>Rev: GCTGACCATCTCGGCTATTT
MAB_2301	Fw: CAGCTCCATCCCATTCCTTATC<br>Rev: TCGGCCTGGACCTTCATA
MAB_2303	Fw: ACATTCTCTGTCCCGATCATTC<br>Rev: GTGCTCTTTACCGACCTCTTC
MAB_2570c	Fw: GGCTCAAGGAAGTAATGGAGAA<br>Rev: GTGTGGCTCCCAATACAGATAG
MAB_2571c	Fw: CTCTTGTTGGCGTGTTGATTAC<br>Rev: CGAACGACGAGATTCCAATGA
MAB_2650	Fw: GAAAGCGGACTCGGGTATTT<br>Rev: TTGGGCTTGTATGTCGGAAG
MAB_3150	Fw: ACTGGTCATCCTGGTGATTG<br>Rev: CATCCGCCTTCGTCATACTT
MAB_3201	Fw: CCGATTCTCGATACCCATTCTC<br>Rev: GTGCTGCTTGATCACTGTTTC
MAB_3562c	Fw: GGGAGTTTGTTCGGGCTATT<br>Rev: GCACCCACCATGACGATAAT
MAB_4240c	Fw: CTCGATGAAGGAGATGGGAAAG<br>Rev: GCTCGATCAACGCCGAATA
MAB_4263	Fw: CTGATTCCGACCTCGATGATG<br>Rev: GCCGATGAAGAAGATGGAGTAG
MAB_4310c	Fw: AAGCCCAACAGCAAGAATTG<br>Rev: GAATACATCGGGCGGTAGATAG
MAB_4382c	Fw: GGGCTATCACACCACCTAT<br>Rev: CGGGTTCATCTTGGCTTTA
MAB_4383c	Fw: GTCATGCATTTACGCACCAAC<br>Rev: CGAAGACCTCATACGTCACAAC
MAB_4704c	Fw: CTCGGCAATCTGGGATTCATAG<br>Rev: CTCTTGGTATCGCCCAGTAAAG
LacZ	Fw: CCAACGTGACCTATCCCATTAC<br>Rev: TTCCTGTAGCCAGCTTTCATC
**Eletrophoretic Mobility Shift Assay (EMSA)**	
Probe 1	Fw: CAC TTC GCC *ATA AGT GGA TTG ACT CTA TCC ACT TTT* ACC CAT AGA (Fluorescein in 5’)<br>Rev :TCT ATG GGT AAA AGT GGA TAG AGT CAA TCC ACT TAT GGC GAA GTG
Probe 2	Fw: CAC TTC GCC **ATG GAC TTC GTG A**C*T CTA TCC ACT TTT* ACC CAT AGA (Fluorescein in 5’)<br>Rev: TCT ATG GGT AAA AGT GGA TAG AGT CAC GAA GTC CAT GGC GAA GTG
Non-specific probe	Fw: ATG GAA CTT GAA GGA CTG ACC GCG TTG (Fluorescein in 5’)<br>Rev: CAA CGC GGT CAG TCC TTC AAG TTC CAT
Probe 3	Fw:**TCT GGA TAA *GC****A AGT GGA TTG ACT CTA TCC ACT* ***TCC* GAA ACC GAC**<br>Rev: GTC GGT TTC GGA AGT GGA TAG AGT CAA TCC ACT TGC TTA TCC AGA

Probe 4	Fw: CAC TTC GCC *ATA AGT GGA T***C***A TCC ACT TTT* ACC CAT AGA (Fluorescein in 5’)<br>Rev: TCT ATG GGT AAA AGT GGA TGA TCC ACT TAT GG C GAA GTG
Probe 5	Fw: CACT TCG CC*A TAA GTG GAT* **ATG CA***T GA*C *TCT C***TG AAG** *ATC CAC TTT T*AC CCA TAG A (Fluorescein in 5’)<br>Rev: TCT ATG GGT AAA AGT GGA TCT TCA GAG AGT CAT GCA TAT CCA CTT ATG GCG AAG TG
Probe 6	Fw: CAC TTC GCC *AT***G G***GT GGA TTG ACT CTA TCC AC***C C***TT* ACC CAT AGA (Fluorescein in 5’)<br>Rev: TCT ATG GGT AAG GGT GGA TAG AGT CAA TCC ACC CAT GGC GAA GTG
Probe 7	Fw: CAC TTC GCC *ATA AGT GG***G C***TG ACT CT***G C***CC ACT TTT* ACC CAT AGA (Fluorescein in 5’)<br>Rev: TCT ATG GGT AAA AGT GGG CAG AGT CAG CCC ACT TAT GGC GAA GTG
Probe 8	Fw: CAC TTC GCC *ATA AG***C A***GA TTG ACT CTA TC***T G***CT TTT* ACC CAT AGA (Fluorescein in 5’)<br>Rev: TCT ATG GGT AAA AGC AGA TAG AGT CAA TCT GCT TAT GGC GAA GTG

[a]Restriction sites are underlined and specified inside brackets.
[b]Mutagenized bases are shown in bold.
[c]Inverted palindromes are shown in italic on the 5’ primer.

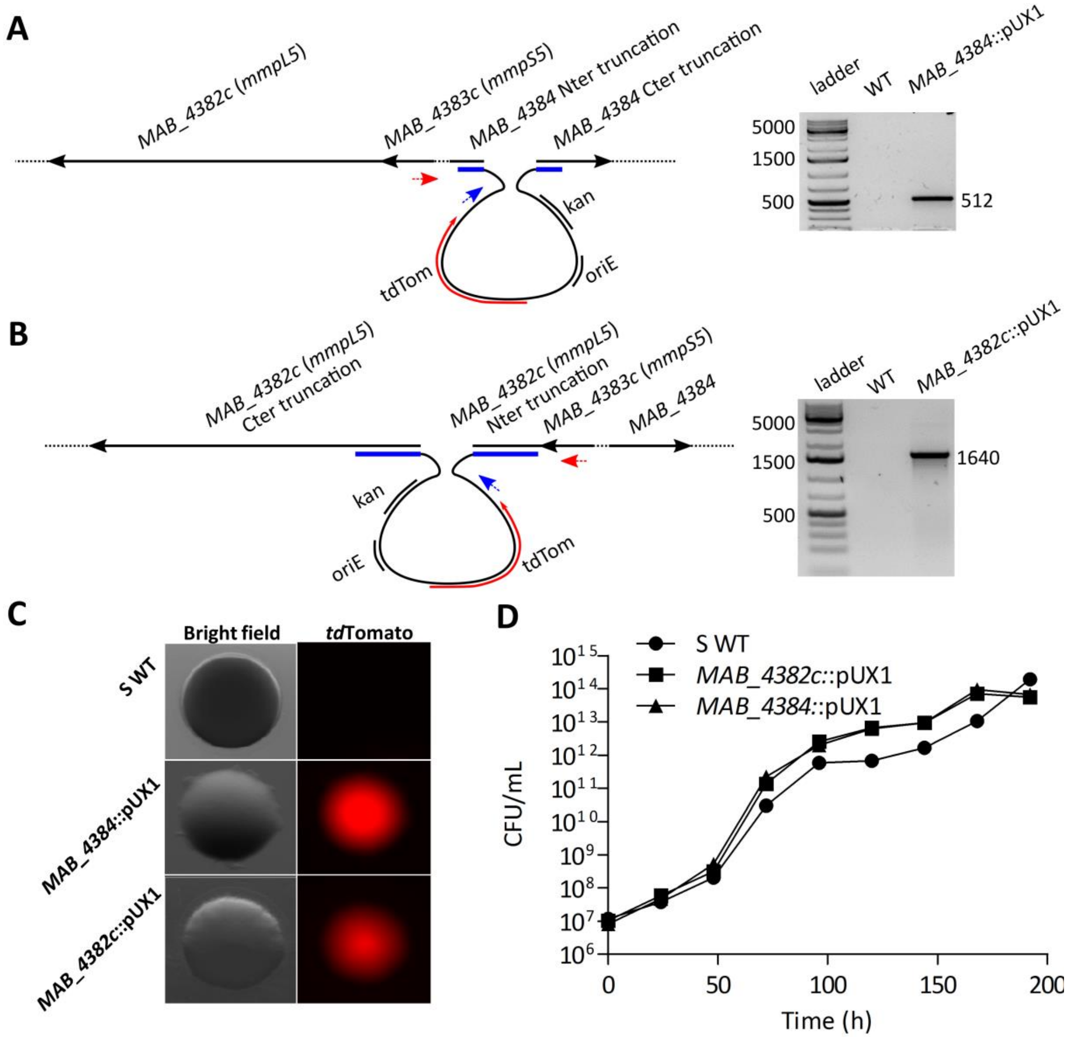

Figure S1. Generation of *M. abscessus* mutants with interruptions in *MAB_4384* and *MAB_4382c* (*mmpL5*). The strategy involves the production of suicide vectors that homologously recombine within targeted genes and carrying a kanamycin cassette as well as a *td*Tomato marker for a rapid screening of transformants having undergone gene disruption (Viljoen et al. 2018). The schematic representation of disrupted *MAB_4384* **(A)** and *MAB_4382c* **(B)** is shown. Proper gene disruption was confirmed by PCR analyses after selection of the red fluorescent colonies **(C)** obtained after transformations. Specific sets of primers were designed to only amplify a band of the expected size in the mutant strains. Blue arrows represent primers that hybridize within the inserted plasmid whereas red arrows correspond to primers only hybridizing within the chromosome. Sizes of the PCR products were analyzed on agarose gels and further sequenced to confirm the gene disruption. **(D)** *In vitro* growth of the parental *M. abscessus* S strain and its *MAB_4384::*pUX1 and *MAB_4382c::*pUX1 derivatives. Bacteria were grown in 7H9 broth supplemented with ADC and 0.05% Tween 80. Growth was monitored by determining the CFU/ml at different time points.

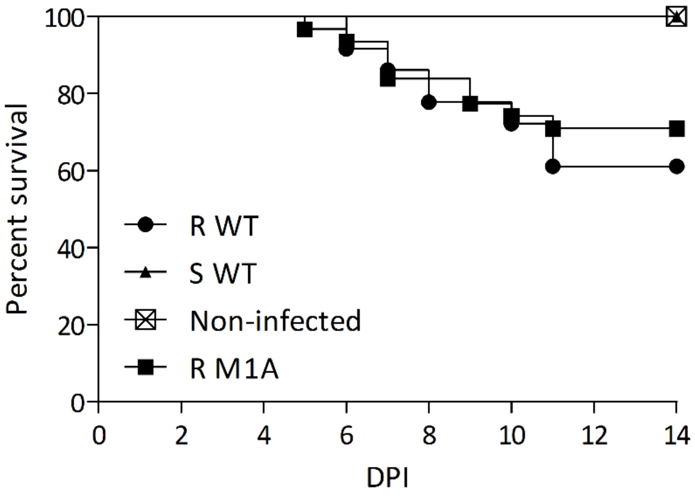

Figure S2. Survival curves of *M. abscessus* strains in zebrafish embryos. Embryos were infected intravenously the wild-type smooth (S) (350 CFU), rough (R) (220 CFU) or MAB_4384(M1A) mutant (280 CFU) strains expressing tdTomato as reported earlier (Bernut et al., 2014, 2015). Groups of uninfected fishes were also included as controls. Dead embryos succumbing from infection were monitored on a daily basis up to 14 days post-infection (dpi). Results are representative of two independent experiments.

All zebrafish experiments were approved by the Direction Sanitaire et Vétérinaire de l'Hérault et Comité d'Ethique pour l'Expérimentation Animale de la région Languedoc Roussillon under the reference CEEA-LR-1145.
